# The utility of CAD in recovering Gondwanan vicariance events and the evolutionary history of Aciliini (Coleoptera: Dytiscidae)

**DOI:** 10.1186/1471-2148-14-5

**Published:** 2014-01-14

**Authors:** Rasa Bukontaite, Kelly B Miller, Johannes Bergsten

**Affiliations:** 1 Department of Entomology, Swedish Museum of Natural History, Box 50007, SE-104 05 Stockholm, Sweden; 2 Department of Zoology, Stockholm University, SE-106 91 Stockholm, Sweden; 3 Department of Biology and Museum of Southwestern Biology, University of New Mexico, Albuquerque, NM 87131, USA

**Keywords:** CAD, Phylogenetic informativeness, Adephaga, Biogeography, Phylogeny, Aciliini

## Abstract

**Background:**

Aciliini presently includes 69 species of medium-sized water beetles distributed on all continents except Antarctica. The pattern of distribution with several genera confined to different continents of the Southern Hemisphere raises the yet untested hypothesis of a Gondwana vicariance origin. The monophyly of Aciliini has been questioned with regard to Eretini, and there are competing hypotheses about the intergeneric relationship in the tribe. This study is the first comprehensive phylogenetic analysis focused on the tribe Aciliini and it is based on eight gene fragments. The aims of the present study are: 1) to test the monophyly of Aciliini and clarify the position of the tribe Eretini and to resolve the relationship among genera within Aciliini, 2) to calibrate the divergence times within Aciliini and test different biogeographical scenarios, and 3) to evaluate the utility of the gene CAD for phylogenetic analysis in Dytiscidae.

**Results:**

Our analyses confirm monophyly of Aciliini with Eretini as its sister group. Each of six genera which have multiple species are also supported as monophyletic. The origin of the tribe is firmly based in the Southern Hemisphere with the arrangement of Neotropical and Afrotropical taxa as the most basal clades suggesting a Gondwana vicariance origin. However, the uncertainty as to whether a fossil can be used as a stem-or crowngroup calibration point for *Acilius* influenced the result: as crowngroup calibration, the 95% HPD interval for the basal nodes included the geological age estimate for the Gondwana break-up, but as a stem group calibration the basal nodes were too young. Our study suggests CAD to be the most informative marker between 15 and 50 Ma. Notably, the 2000 bp CAD fragment analyzed alone fully resolved the tree with high support.

**Conclusions:**

1) Molecular data confirmed Aciliini as a monophyletic group. 2) Bayesian optimizations of the biogeographical history are consistent with an influence of Gondwana break-up history, but were dependent on the calibration method. 3) The evaluation using a method of phylogenetic signal per base pair indicated Wnt and CAD as the most informative of our sampled genes.

## Background

The tribe Aciliini Thomson, 1867 belongs to the subfamily Dytiscinae, comprising most of the larger diving beetles within Dytiscidae. Aciliini presently include 69 described species in seven genera [1]. Adults have generally good flight capability and can be found in both temporary and permanent water bodies [2-4]. They are distributed worldwide and mostly inhabit standing waters like pools, ponds, lakes, swamps and bogs [1,3,4]. Both larvae and adults are predatory on smaller aquatic invertebrates and the larvae have a characteristic arched body shape with a small narrow head.

**Figure 1:**
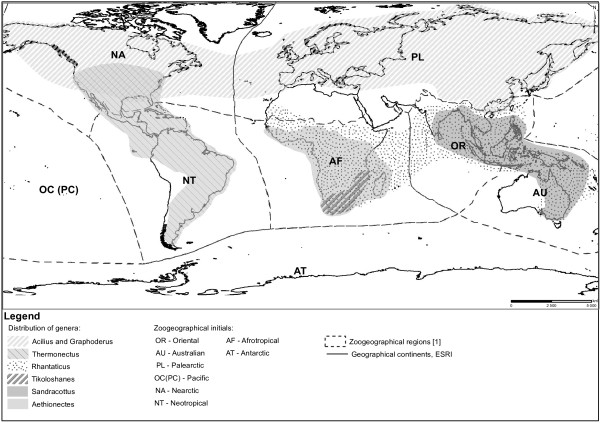
Approximate geographical distribution of Aciliini genera.

The distribution of the tribe covers all main zoogeographical regions except Antarctica, but distribution of the genera suggests an ancient origin (Figure 1). *Thermonectus* Dejean, 1833 is the most speciose genus of the tribe (with eighteen species) and occurs in the Neotropics and the Nearctic north to the Canadian border [2]. The genera *Acilius* Leach, 1817 and *Graphoderus* Dejean, 1833, which each include more than ten species, are restricted to the Holarctic. *Aethionectes* Sharp, 1882 is a small Afrotropical genus with eight species, one of which is restricted to Madagascar. *Sandracottus* Sharp, 1882 occurs in the Australasian region. *Rhantaticus* Sharp, 1882 is a widespread genus with currently a single recognized species *R. congestus* (Klug, 1833) occurring throughout the Afrotropical, south Palearctic, Oriental and Australian regions. However, preliminary studies suggest that it is a species complex in need of revision. Finally, the most recently added genus *Tikoloshanes* Omer-Cooper, 1956 includes the single species *T. eretiformis* Omer-Cooper, 1956. It is rarely collected, never in great numbers, and only known from southeastern Africa. *Tikoloshanes* share similarities with the tribe Eretini Crotch, 1873 as the color and shape are very similar to those of *Eretes* Laporte, 1833, but the genus was proposed as a new genus within Aciliini, close to *Aethionectes*[5]. *Tikoloshanes* has never been included in a phylogenetic analysis using molecular data. The tribe Aciliini has not previously undergone any major revision or phylogenetic analysis, but the genus *Acilius* was recently revised [3] and its evolutionary history inferred with molecular and morphological data [6].

Erichson [7] referred species today in *Graphoderus*, *Sandracottus* and *Thermonectus* to *Hydaticus* until 1833 when Dejean [8] recognized *Thermonectus* and *Graphoderus* as separate genera. Aubé [9] transferred *Thermonectus* to a subgroup of *Acilius* and *Graphoderus* to a subgroup of *Hydaticus*. Régimbart [10] was the first author who suggested a delimitation of *Acilius*, *Graphoderus* and *Hydaticus* similar to the one used today based on characters of the metasternal wing and metatibial spurs, but *Thermonectus* and *Sandracottus* were included in *Graphoderus*. A few years later, Sharp [11] formalized this view, erected the tribes Thermonectini (=Aciliini) and Hydaticini, and recognized the six genera of Aciliini which are generally accepted today (except the more recently described *Tikoloshanes*). Hence, since the early classification there has been two alternative hypotheses. First, the genus *Acilius* has been regarded as the most divergent of the genera based on morphology, especially that of the females and the wide body shape. This is reflected in, for example, Erichson’s [7] classification, with *Graphoderus*, *Sandracottus* and *Thermonectus* grouped with each other near *Hydaticus*. An alternative hypothesis is the view of Aubé [9] that there are similarities between *Acilius* and *Thermonectus* to the exclusion of the other genera. These hypotheses are mutually exclusive if similarity is to be interpreted as evidence of sister group relationships, although they were erected prior to the development of a cladistic theory and could reflect similarity based on shared plesiomorphic character states.

The evolutionary relationships of Aciliini genera have never been analyzed in detail with modern phylogenetic methods. Only a handful of studies with different foci have included a few representatives in phylogenetic analyses. Miller’s [12] study on Dytiscinae was based on adult morphology and recovered *Graphoderus* as a group sister to the rest of Aciliini, and *Acilius* and *Thermonectus* as sister clades in agreement with Aubé [9]. In a later morphological study, with a larger taxon sampling for all of Dytiscidae and with a focus on the female reproductive tract, Miller [13] included all known genera of Aciliini. However the genera *Aethionectes, Rhantaticus, Sandracottus* and *Tikoloshanes* were merged into a single terminal as they scored identically for all morphological characters used. Again, *Acilius* and *Thermonectus* came out as sister taxa [13].

In a molecular study Ribera et al. [14] included single representatives of five of the seven Aciliini genera and arrived at a somewhat different intergeneric topology. In their analysis *Acilius* instead came out as sister to *Graphoderus,* and *Thermonectus* was placed as sister to this clade. Also *Rhantaticus* and *Sandracottus* were identified as sister groups and, together, sister to the rest of Aciliini. Moreover they recovered Eretini nested inside Aciliini suggesting the tribe may be paraphyletic. Only the first conclusion was strongly supported with posterior probability of > 0.95. However their main focus was on the phylogeny of supra-generic groups of Dytiscidae based on two mitochondrial and two nuclear genes, including five species of Aciliini and two species of Eretini. Eretini, which includes only the genus *Eretes*, has been proposed to be the sister tribe to Aciliini, since it shares similar setae on the metafemur which are apically bifid and arranged in a strongly oblique row [12,15]. The larvae also share a unique shape, an erratic shrimp-like swimming-escape behavior and two pairs of large camera-like stemmata which may be an additional apomorphy for Aciliini and Eretini [16]. The prospect of a paraphyletic Aciliini with Eretini nested inside was quite surprising, but tantalizingly suggests the possibility that the enigmatic *Tikoloshanes eretiformis* is, indeed, closely related to Eretini.

A dated phylogenetic hypothesis with robust support is needed in order to test whether the intergeneric relationships in the group, and the age of the group, supports a Gondwana break-up origin. Both the hypotheses outlined above contradict such an origin. *Acilius* basal would infer a Holarctic origin of the group and an *Acilius* + *Thermonectus* sister group relationship would imply a much more recent Holarctic–Neotropical connection and diversification. Neither is consistent with a Gondwana break-up origin, which would require basal positions of southern hemisphere clades. Fossils in the tribe are very sparse and of no direct help to place the origin of the group in the Cretaceous as all are of younger Neogene or late Paleogene ages. However, the use of relaxed clock models and Bayesian frameworks to include the calibration uncertainty as prior distributions can make use of such fossil to derive age span estimates for the basal nodes in a well-supported tree.

The search for new nuclear coding genes for phylogeny reconstruction in different target groups is ongoing [17]. Coleoptera does not contain any high priority model organisms except perhaps the flour beetle, *Tribolium castaneum*, but its complete genome was not entirely sequenced until 2008 [18], which is one reason the selection of easily amplifiable single-copy nuclear genes in beetles has lagged behind many other groups of organisms. The nuclear protein-coding gene CAD, also known as rudimentary, was for the first time introduced to the insect systematics community by Moulton and Wiegmann [17]. Since then it was successfully used in e.g. Diptera [17], Hymenoptera [19-21], Neuropterida [22], Trichoptera [23] and Lepidoptera studies [24]. CAD performed well both separately and in combination with other genes as evaluated by Johanson and Malm [23]. Some studies to date have used CAD in Coleoptera [25-34], mainly in Carabidae, Scolytinae and Silphidae but none have yet used CAD in Adephagan water beetle (Hydradephaga) phylogenetics.

The CAD gene encodes three enzymes that catalyze pyrimidine biosynthesis: carbamoyl phospate synthetase (CPS), aspartate transcarbamylase (ATC) and dihydroorotase (DHO) and with a combined total length of about 6.6 kb. Recent studies on Braconidae by Sharanowski et al. [35] demonstrated that the CPS region contains a high number of parsimony informative sites. Wild and Maddison [27] suggested CAD to be the highest performing gene fragment for both shallow and deep phylogenetics among eight evaluated nuclear genes in beetles and designed several primer pairs for a 2020 bp CPS region, free of introns in most taxa of beetles.

This study is the first comprehensive phylogenetic analysis focused on the tribe Aciliini and it is based on eight gene fragments. The aims of the present study are: 1) to test the monophyly of Aciliini, clarify the position of the tribe Eretini and to resolve the relationship among genera within Aciliini 2) to calibrate the divergence times within Aciliini, reconstruct ancestral distributions and thereby test the hypothesis of a Gondwana vicariance influence, and 3) to evaluate the utility of CAD for phylogenetic analysis in Dytiscidae.

## Methods

### Taxon sampling

Taxon sampling was focused on assembling the highest species representation as possible for the tribes Aciliini and Eretini. Our ingroup comprised species from all seven genera of Aciliini (61% of the known Aciliini species): *Acilius* (12 species, 92%), *Aethionectes* (2 species, 25%), *Graphoderus* (10 species, 91%), *Rhantaticus* (1 species, 100%), *Sandracottus* (5 species, 31%), *Thermonectus* (11 species, 58%), *Tikoloshanes* (1 species, 100%), and all four species of Eretini represented by the single genus *Eretes*. Our taxa cover all six zoogeographic regions: Nearctic (*Acilius*, *Graphoderus*, *Thermonectus*), Neotropical (*Thermonectus*), Palearctic (*Acilius*, *Graphoderus*, *Sandracottus, Rhantaticus*), Afrotropical (*Aethionectes*, *Rhantaticus*, *Tikoloshanes*), Oriental (*Rhantaticus, Sandracottus*) and Australian (*Sandracottus, Rhantaticus*) (see Additional file 1: Table S1).

Since *Rhantaticus congestus* is suspected to actually represent a species complex we included several specimens of *Rhantaticus* from different regions. As outgroups to root the tree we used representatives from Hydaticini, which are thought to be the closest relatives [12], and Dytiscini.

### DNA extraction, amplification and sequencing methods

For most specimens whole genomic DNA was extracted using Qiagen DNEasy kit (Valencia, California, USA) and the animal tissue protocol. Thoracic muscle tissue was removed from large specimens for extraction. A few small specimens were extracted by removing the abdomen and placing the remaining portion of the specimen in extraction buffer for incubation. Dissected specimens and whole extracted specimens were retained for vouchering in the Museum of Southwestern Biology Division of Arthropods (MSBA, K.B. Miller, curator). Some DNA was extracted from legs of ethanol-preserved or dry-mounted material using the DNEasy DNA extraction kit (Qiagen, Valencia, CA) following the manufacturer’s recommendation, except for that 20 μl of 1 molar DTT (dithiothreitol) was added during the lysis stage. The GeneMole robot and MoleStrips™ DNA Tissue (Mole Genetics) was used for isolation of DNA from some of the samples with an elution volume of 100 μl. After extraction, legs were returned to the vouchers for archiving.

Eight gene fragments were selected to infer the phylogeny and divergence times. These included three nuclear protein-coding genes, histone 3 (H3), wingless (Wnt) and rudimentary (CAD, a part of CPS), two ribosomal genes, mitochondrial 16S and nuclear 28S, and the mitochondrial protein-coding genes cytochrome c oxidase subunit I (COI) and II (COII). COI was sequenced in two non-overlapping fragments. Primers used for amplification and sequencing were derived from several sources, but also new primers were designed for some taxa (see Additional file 2: Table S2).

Most of the DNA fragments were amplified using “Ready-to-go™” PCR Beads from Pharmacia Biotech following the manufacture’s standard protocols. Each 25 μl reaction contained 1 μl of 10 μM primer pair mix (×2), 2 μl of DNA template and 21 μl of water. The thermal cycling profile for “Ready-to-go” PCR was 94°C for 5 min, followed by 40 cycles of 94°C 30 s, 56-50°C for 30 sec, 72°C for 1 min and finally 72°C for 8 min. Product yield, specificity of amplification and contamination were investigated using agarose gel electrophoresis. PCR products were purified using ExoFast Cleanup mix (Fermentas) and cycle sequenced using the same primers as in the PCR. For sequencing reactions the ABI BigDye Terminator kit ver. 3.1 kit (Applied Biosystems) was used. Each sequencing reaction mixture included 1 μl of BigDye™ (Applied Biosystems), 1 μl of 1,6 μM primer and 2-4 μl of PCR product. The sequence cycling profile was 95°C for 1 min and then 25 cycles of 95°C 30 sec, 50°C 15 sec and 60°C 4 min. Sequencing products were purified using the DyeEx 96 kit and fragments were analyzed on an ABI377xl analyzer from Applied Biosystems at the Molecular Systematics Laboratory, Swedish Museum of Natural History.

Some fragments were amplified using PCR with TaKaRa Amplitaq (Applied Biosystems, Foster City, CA, USA) on an Eppendorf Mastercycler ep gradient S thermal cycler (Eppendorf, Hamburg, Germany). Amplification conditions were similar to those used by Miller et al. [6,36,37]. Products were purified using ExoSAP-IT (USB-Affymetrix, Cleveland, OH, USA) and cycle sequenced using ABI Prism Big Dye (v3.1; Invitrogen, Fairfax, VA, USA) using the same primers as for amplification. Sequencing reaction products were purified using Sephadex G-50 Medium (GE Healthcare, Uppsala, Sweden) and sequenced using an ABI3130xl Genetic Analyzer (Applied Biosystems, Foster City, CA, USA) in the Molecular Biology Facility at the University of New Mexico.

Gene regions were sequenced in both directions. Resulting sequence data were examined and edited using the program Sequencher 4.10 (Gene Codes Corporation). The forward and reverse primers were trimmed from the beginning and end of each sequence. Sequences are submitted to NCBI GenBank under accession numbers KF978819-KF979120.

For some taxa we did not retrieve full length fragments, probably due to DNA degradation especially for the dry-pinned specimens. Sequences from all eight gene fragments were obtained from specimens when possible and missing data represented 11% at the gene fragment level. DNA sequences were aligned with MUSCLE [38] in MEGA 5 [39] using the default settings. The protein-coding genes contained no gaps except for CAD which had a 3, 6 or 9 bp long indel at one position. The final alignment of the eight gene segments included 6095 bp and all gaps were treated as missing data in the analyses. Data tables and matrices were created with Voseq 1.4.4 [40].

### Partition finder

To determine the best-fit partitioning scheme of molecular evolution for our dataset we used PartitionFinder V1-1.0.1 [41]. During analyses branch lengths were unlinked to allow the program to estimate them independently for each subset. The best model, among the ones available in MrBayes 3.2 [42], was searched for under the greedy search algorithm based on the Bayesian Information Criterion (BIC) model metric. Based on BIC scores for each partition, GTR + I + G or HKY + G model were the best-scoring models, followed closely by the SYM + I + G and HKY + I + G models. We used the partitioning scheme and among-site rate variation suggested by PartitionFinder but instead of selecting one substitution model *a priori*, we used reversible-jump MCMC to allow sampling across the entire substitution rate model space [43] (see Additional file 3: Table S3). Apart from using the partitioning scheme suggested by Partition Finder, we also tested partitioning our data following a commonly suggest scheme with six partitions [37]: 1) 1^st^ and 2^nd^ codon position of mitochondrial protein-coding genes, 2) 3^rd^ codon position of mitochondrial protein-coding genes, 3) 1^st^ and 2^nd^ codon position of nuclear protein-coding genes, 4) 3^rd^ codon position of nuclear protein-coding genes, 5) mitochondrial ribosomal genes (16S), 6) nuclear ribosomal genes (28S).

### Phylogenetic analyses

We used Bayesian inference to estimate the phylogenetic relationship among taxa. Four taxa were used as outgroups: *Hydaticus aruspex* Clark, 1864*, Hydaticus transversalis* Pontoppidan, 1763*, Hydaticus leander* Rossi, 1790 and *Dytiscus verticalis* Say, 1823. Hydaticini is considered to be the closest relative to Aciliini + Eretini among the outgroups included in this study [12,14]. Data were first examined by analyzing the different gene regions separately. The final phylogenetic analysis was conducted on the combined data matrix of all eight gene fragments.

The partitioned data set was analyzed by Bayesian methods using Metropolis-coupled Markov Chain Monte Carlo (MCMC) in MrBayes 3.2 [42]. All partitions were unlinked allowing each partition to have its own set of parameters. The analysis was run with four chains for 10 000 000 generations with a sample frequency of 1000 and a burn-in value of 25%. The parameter estimations were checked in Tracer v 1.5. [44].

All analyses were repeated twice to ensure that the final trees converged to the same topology and convergence of runs was monitored with the statistics provided by MrBayes 3.2. The final tree was visualized in FigTree 1.3 and annotated with Adobe Illustrator CS5.

To ensure that the MCMC analyses had run long enough such that tree topologies were sampled in proportion to their true posterior probability distribution, we used AWTY [45,46] for graphical visualization of MCMC convergence.

### Dating analysis

A calibrated tree was obtained using the topology retrieved from MrBayes in a Bayesian MCMC analysis with BEAST 1.7.5 [44]. We used the same partitions as in the MrBayes analyses. Since Beast does not allow the reversible jump MCMC the General Time Reversible substitution model with gamma and invariant sites (GTR + Γ + I) was selected. The uncorrelated lognormal clock was used to relax the equal rates constraint across the tree that a strict clock enforces [47].

Our primary calibration point was based on the fossil of *Acilius florissantensis* Wickham, 1909. The fossil is from the Florissant formation in Colorado, USA, which occurs on the boundary between Oligocene and Eocene, and has been dated to 34.07 +/- 0.10 Ma [48]. Based on the description of the fossil it is possible to either associate the fossil with the monophyletic Nearctic clade of *Acilius*[6] or to the whole genus. The narrower body-shape is in agreement with the Nearctic clade (as is the location of the formation), and Wickham [49] mainly compared the fossil with Nearctic *A. semisulcatus* Aubé, 1838. But that body-shape is also found in one of the Palearctic species (*A. duvergeri* Gobert, 1874) and it is ambiguous if this is the apomorphic or plesiomorphic state in the genus. The fossil is a ventral impression of a male, whereas a dorsal female impression could have been more informative. Because of this uncertainty in placement we tested the effect of using it both as a stem-node constraint of *Acilius* and as a crown-node constraint of *Acilius*, the latter appropriate if the fossil can be assigned to the Nearctic clade. The prior on the age of the node was set to a lognormal distribution with an offset at 34,0 Ma (mean = 7, Std = 5,0), which gave a distribution with median at 40 Ma and 95% of the prior distribution between 36 and 50 Ma. As a fossil only represents a minimum age of a node and the real age is likely to be some amount older followed by a declining tail of probability further back, this lognormal prior captures more or less our prior beliefs [50,51].

We also performed the same analyses using an exponential prior (offset = 34 Ma, mean = 5, 95% of the distribution between 34 and 52 Ma) instead of a lognormal prior (see [50] for a discussion of the use of exponential versus lognormal distribution to model fossil calibration uncertainty). The results were very similar compared to the difference of using the fossil on either the stem or crown node, why only the lognormal and the node of calibration will be discussed further.

Proposals for updating the topology in the MCMC chain were inactivated so that only branch lengths were estimated in this analysis. The analysis was run for 30 000 000 generations sampled every 3000 generations with a burn-in value of 10%. We used TreeAnnotator to calculate the maximum clade credibility tree and visualized it in FigTree.

### Biogeographic analyses

We used Bayesian Binary Method (BBM) implemented in RASP (Reconstruct Ancestral State in Phylogenies) [52] to reconstruct the ancestral areas of Aciliini. The current distribution areas of species (Figure 1) were obtained from Nilsson [1] and coded as follows: A (Nearctic), B (Neotropical), C (Afrotropical), D (Oriental), E (Australian), F (Palearctic). The analysis was conducted on the dated tree based on the primary calibration point as a crown group constraint of *Acilius* with the lognormal prior distribution. The outgroup taxa were removed from the analyses, since the model assigns virtual outgroups to the phylogenetic tree prior to the start of the analyses.

The four MCMC chains were run simultaneously for 10 000 000 generations. The state was sampled every 1000 generations. State estimation was run under the F81 + G model for the BBM analysis with wide root distribution to allow assigned outgroup to occur in all areas occupied by the ingroup. This is the most complex model implemented in RASP, with properties expected to yield realism to reconstruct ancestral distribution. The maximum number of areas for this analysis was kept to six.

### Phylogenetic signal and saturation

We applied the phylogenetic signal method of Townsend [53,54], modified to exclude the normalization equation, to determine the Phylogenetic Informativeness (PI) for each gene fragment and each codon position in our dataset. We followed the procedure described by Malm et al. [19] and Klopfstein et al. [55] and calculated site rates in HYPHY 2.1.2 [56], derived from an ultrametric tree with branch lengths proportional to time. PI values were estimated in R (http://www.r-project.org), which allowed us to visualize the evolutionary rates of each codon position over the evolutionary time of our phylogeny [19,53]. We also estimated PI average for each gene codon position by dividing it by gene fragment length for comparison among the genes.

Saturation plots for each gene and codon position were produced by retrieving pairwise uncorrected p-distances, and plotting them against inferred branch-length distances on the tree with the highest likelihood in the tree sample from the Bayesian MCMC analysis [55].

## Results

### Gene sequence variation

The combined and aligned data set included 6095 bp of DNA sequences. The longest fragments were obtained from CAD, 2008 bp, followed by COI 3′end (821 bp), COII (684 bp), COI 5′end (671 bp), 28S (670 bp), Wnt (466 bp), 16S (447 bp) and H3 (328 bp). Length variation in gene sequences obtained during this study was minimal. There were no introns identified in the alignments but we found a highly variable section within the first 150 bp from the 5′ end of the CAD fragment.

**Figure 2:**
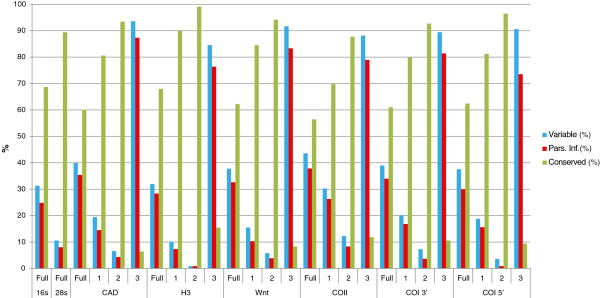
Proportion of variable, conservative and parsimony informative sites for each gene and each codon position.

The combined dataset included 3926 conservative sites and 1862 were parsimony-informative. The proportion of variable sites in nuclear and mitochondrial protein-coding genes were similar (28-38%), while for the ribosomal genes 16S and 28S it was 25 and 8%, respectively. The 3^rd^ codon position among all protein coding genes was the most parsimony informative, about 80% of all characters for each gene (Figure 2). Partition Finder divided the dataset into four partitions: 1) 16S, 2) 28S, 1^st^ and 2^nd^ codon position of mitochondrial and nuclear genes; 3) nuclear genes 3^rd^ codon position; 4) mitochondrial genes 3^rd^ codon position (see Additional file 3: Table S3).

### Phylogenetic relationships

**Figure 3:**
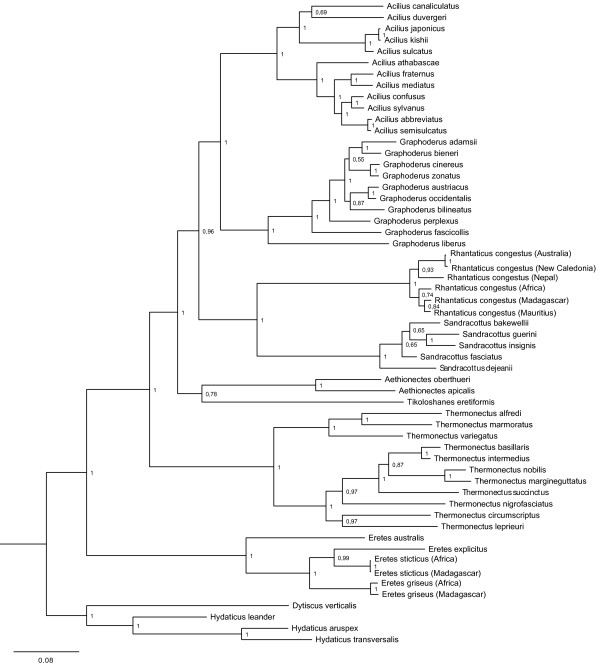
**Phylogeny from Bayesian analysis of all eight combined gene fragments.** Support values next to the nodes are Bayesian posterior probabilities.

The Bayesian analysis of the combined data resulted in a well-resolved and overall highly supported phylogeny (Figure 3). The ingroup Aciliini + Eretini was strongly supported (posterior probability, pp = 1.0) and each tribe was resolved as monophyletic with high support (pp = 1.0). In Eretini, the Australian *Eretes australis* (Erichson, 1842) was recovered as sister to the remaining three taxa in *Eretes* (pp = 1.0). In Aciliini, all six genera were recovered as monophyletic with high support (pp = 1.0).

The genus *Thermonectus* was identified as sister group to the rest of Aciliini with high support (pp = 1.0), and was divided into two main clades. The two Afrotropical genera, *Aethionectes* and *Tikoloshanes,* were recovered as sister clades with moderate support (pp = 0.78) and basal to the rest of Aciliini excluding *Thermonectus* (pp = 0.96).

The remaining Aciliini was divided into two main clades, a Holarctic clade (*Acilius* + *Graphoderus*) and an Australasian-Afrotropical clade (*Rhantaticus* + *Sandracottus*), both with high support (pp = 1.0). The internal resolution within the genera was fully resolved, but, in some cases, especially within *Sandracottus,* with weak support. *Rhantaticus*, probably representing a species complex, was divided into an Afrotropical and an Australasian clade.

The Holarctic clade consisted of *Graphoderus* (pp = 1.0) and *Acilius* (pp = 1.0) with all internal branches highly supported except three. In *Graphoderus* the Nearctic species *G. liberus* was sister to the rest of *Graphoderus* and two other Nearctic taxa form the following consecutive branches, strongly suggesting a Nearctic origin for the genus. The genus *Acilius* was highly supported (pp = 1,0) and divided into two main clades, a Nearctic (pp = 1.0) and a Palearctic (pp = 1.0).

The alternative partitioning scheme with six partitions resulted in an identical intergeneric topology, and only differed at one position within *Thermonectus* and at one position within *Graphoderus*. The sister group relationship of *Tikoloshanes* and *Aethionectes* which had only moderate support in the four partition analysis was given stronger support in the six partition analysis (pp = 0.98).

**Figure 4:**
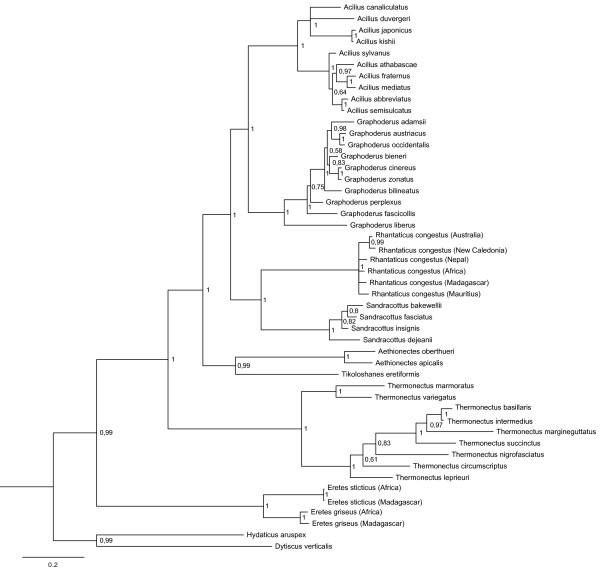
**Phylogeny from Bayesian analysis of the single CAD gene fragment.** Support values next to the nodes are Bayesian posterior probabilities.

To explore the contribution of CAD we ran separate analyses with CAD alone and all data except CAD, based on the same model of evolution described above. CAD alone, which makes up 31% of the entire dataset (35.5% of parsimony informative sites), resulted in a topology identical to the combined analysis in terms of tribal and intergeneric resolutions (Figure 4). The support values among genera were even higher for some nodes. However, species resolutions in some genera were lower. For example, Palearctic species in the genus *Acilius* and resolution within the *Rhantaticus* complex collapsed into polytomies.

**Figure 5:**
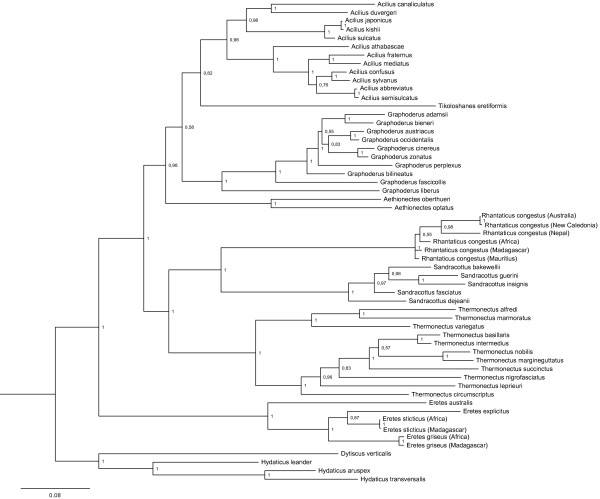
**Phylogeny from Bayesian analysis of all combined gene fragments, but excluding CAD.** Support values next to the nodes are Bayesian posterior probabilities.

Analysis of all data except CAD resulted in a different topology (Figure 5). The intergeneric resolution conflicted with the resolution from CAD alone and from the combined dataset. Aciliini was divided into two main clades: 1)–*Thermonectus* was grouped as sister to *Rhantaticus* + *Sandracottus*, 2)–*Aethionectes* was identified as sister group to the genera *Graphoderus*, *Acilius* and *Tikoloshanes. Tikoloshanes* was unexpectedly nested inside an otherwise Holarctic clade (pp = 0.81).

### Time of divergence

**Figure 6:**
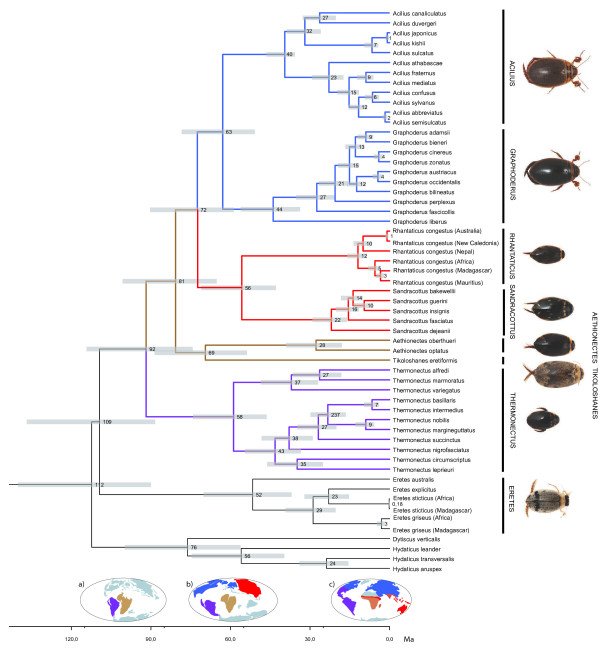
**Timetree of Aciliini.** Time-calibrated Bayesian phylogeny obtained with BEAST based on the fossil *Acilius florissantensis* used as a crown-node constraint on the genus *Acilius*. Horizontal bars at the nodes indicate 95% highest posterior density intervals for the age of the corresponding node. Maps (a-c) at scale bar represent the position of continents at the time (modified from [61]), colored following hypothetical ancestral distributions of the clades obtained from RASP. Map **(a)**: ancestral distribution of *Thermonectus* in South America and of *Tikoloshanes + Aethionectes* in Africa. Map **(b)**: ancestral distribution of *Graphoderus* + *Acilius* in Euramerica and of *Sandracottus* + *Rhantaticus* in Asiamerica appears. Map **(c)**: current distribution of genera (*Tikoloshanes*, *Aethionectes* and *Rhantaticus* overlap in Africa).

The estimation of divergence time using the fossil as a crown-node constraint on *Acilius* suggested congruent ages for the basal nodes with relevant Gondwana break-up ages (Figure 6). The basal divergence of Aciliini and Eretini took place in the mid-Cretaceous around 109 Ma (95% highest posterior density = 137-88 Ma). The first branching event within Aciliini was estimated at 92 Ma (114-74 Ma) and involved the divergence of Neotropical *Thermonectus* from the rest of Aciliini. The second main branching event was the split between Afrotropical genera *Aethionectes* and *Tikoloshanes* and the ancestor of the remaining Aciliini (excluding *Thermonectus*) about 81 Ma (101-65 Ma). *Aethionectes* and *Tikoloshanes* diverged at 69 Ma (88-54 Ma), *Graphoderus* and *Acilius* at 63 Ma (78-51 Ma), and *Rhantaticus* and *Sandracottus* at 56 Ma (71-43 Ma).

Using the fossil calibration as an *Acilius* stem-node constraint gave significantly younger ages not congruent with Gondwana vicariance events. For example the split between Neotropical *Thermonectus* and the rest of Aciliini was estimated at 58 Ma (70-49 Ma), *Graphoderus* and *Acilius* at 40 Ma (46-36 Ma), and *Rhantaticus* and *Sandracottus* at 35 Ma (43-28 Ma).

### Biogeographical analysis

**Figure 7:**
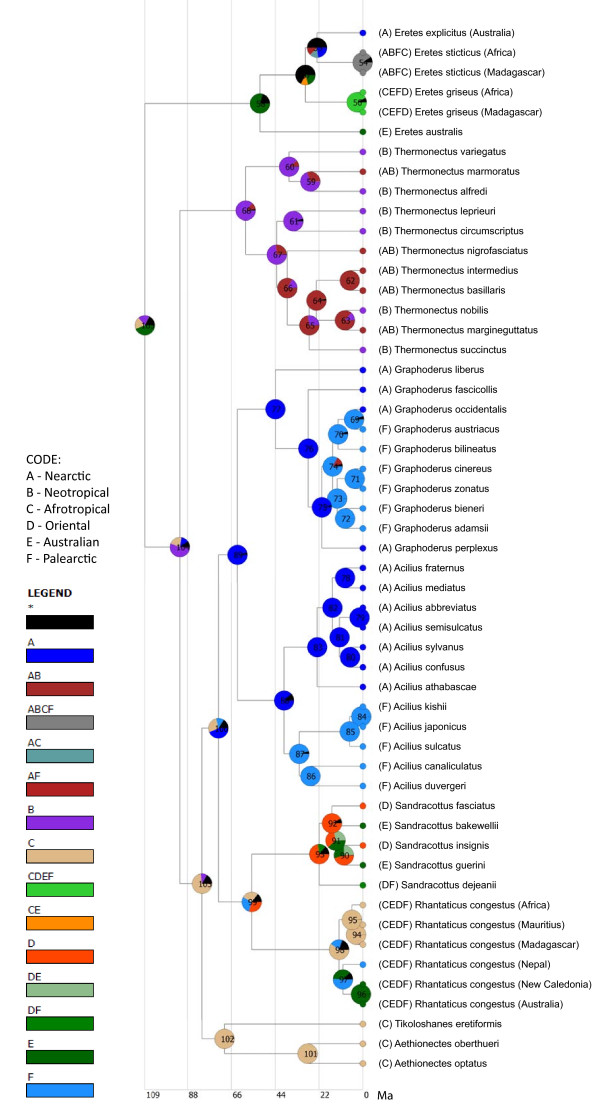
**Ancestral distribution inferred from Bayesian analysis with RASP.** Pie charts represent ancestral distributions as probabilities coded as follows: A (Nearctic), B (Neotropical), C (Afrotropical), D (Oriental), E (Australian), F (Palearctic).

The biogeographical analysis favoured an Neotropical (59%) over an Afrotropical (23%) origin of the tribe Aciliini (Figure 7). So optimized, *Thermonectus* originated in South America and later dispersed to the Nearctic region with is in line with the diversity being greatest in the Neotropical part of the New World. However, the ancestral region for Aciliini excluding *Thermonectus* remained concentrated in the Afrotropical region (80%) where *Aethionectes* and *Tikoloshanes* are found. The deepest divergence within Aciliini is hence between an ancestor in South America and an ancestor in Africa, consistent with a Gondwana vicariance event. This was followed by a series of dispersals to the Palearctic, Oriental, Nearctic and Australian regions during the Cenozoic. The ancestor of the Holarctic *Graphoderus* + *Acilius* was optimized to be of Nearctic origin (91%) with later dispersals to the Palearctic region within each genus independently. The ancestral distribution of the ancestor of Eretini was inferred to most likely be the Australian region (50%).

### Phylogenetic informativeness

**Figure 8:**
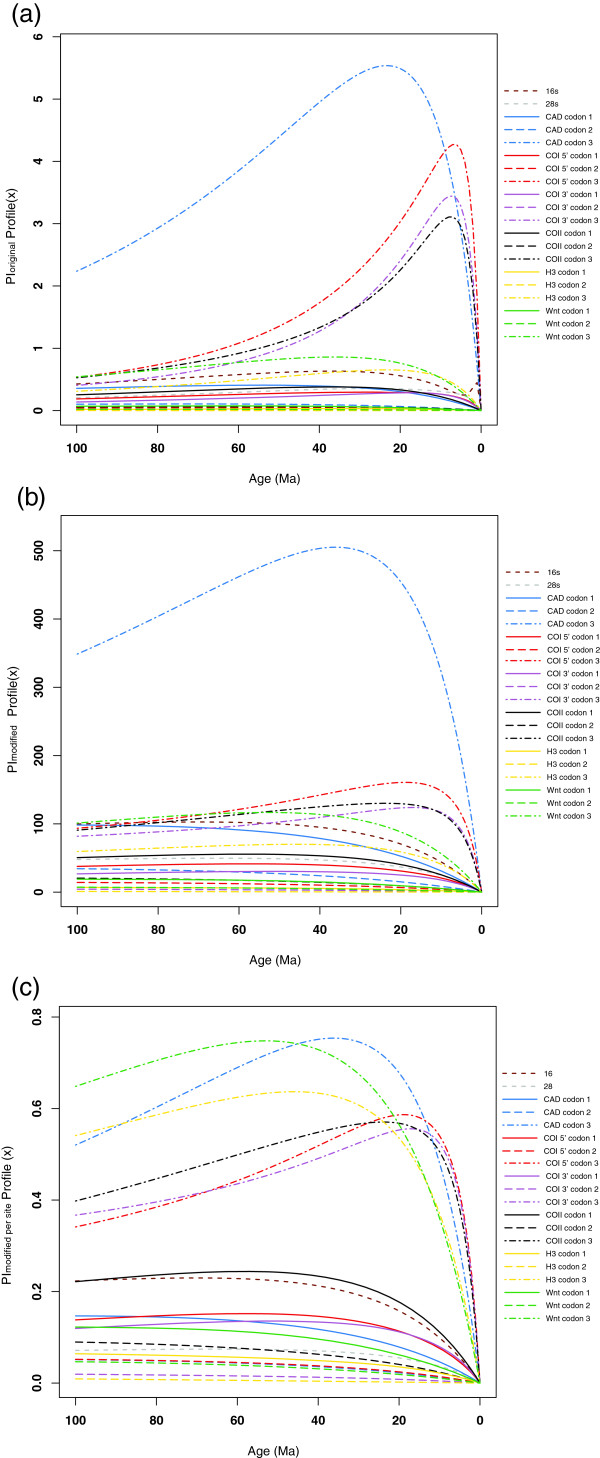
**Phylogenetic Informativeness (PI) plots for all data partitions. (a)** PIoriginal, **(b)** PImodified, **(c)** PIaverage-modified. These analyses differ in the use of a normalization step in PIoriginal, which is removed in PImodified and PIaverage-modified. The latter differ from PImodified in averaging PI values over the gene length. The X-axis denotes time from 100 Ma to present, while the Y-axis denotes Phylogentic Informativeness.

The phylogenetic informativeness (PI) curves, which attempt to visualize phylogenetic information over time for a given dataset, showed high peaks at ages younger than 20 Ma for the 3^rd^ codon positions of all mitochondrial protein-coding genes (Figure 8a-c). This indicates a partition most informative for the radiation within genera. The 3^rd^ codon positions of the nuclear protein-coding genes showed the highest PI values between 35-50 Ma and a trailing slow decline over deeper time. For the 1^st^ and 2^nd^ codon positions PI increased slowly with time until 30-50 Ma, and was overall lower for both nuclear and mitochondrial genes and more extended over time.

The original Townsend’s PI profile (Figure 8a) gave more narrow peaks than the Modified Townsend’s PI profile (Figure 8b), suggesting higher level of “noise” in the data, which can mislead phylogenetic analysis [56]. In the latter analysis without normalization (Figure 8b) these peaks were more extended, relatively lower, and slower evolving positions surpass faster ones at deeper time-scales (for example, CAD 1^st^ codon position shows higher PI at 95 Ma than 3^rd^ codon positions of all mitochondrial protein-coding genes). Third codon position of CAD sticks out as the most informative partition in the dataset. This is partly because this gene fragment is three times longer than the other genes. However average-modified Townsend’s profile displays a PI plot where length differences have been taken into account for comparison across genes and codon positions (Figure 8c). This probably gives the fairest view of comparing partitions and it clearly shows that, 1) most information is in the 3^rd^ codon positions and 2) nuclear 3^rd^ codon positions are superior to mitochondrial per aligned base at ages older than 25 Ma. CAD reached the highest PI peak at about 35 Ma, but was surpassed by Wnt at deeper time-scales.

### Gene saturation

**Figure 9:**
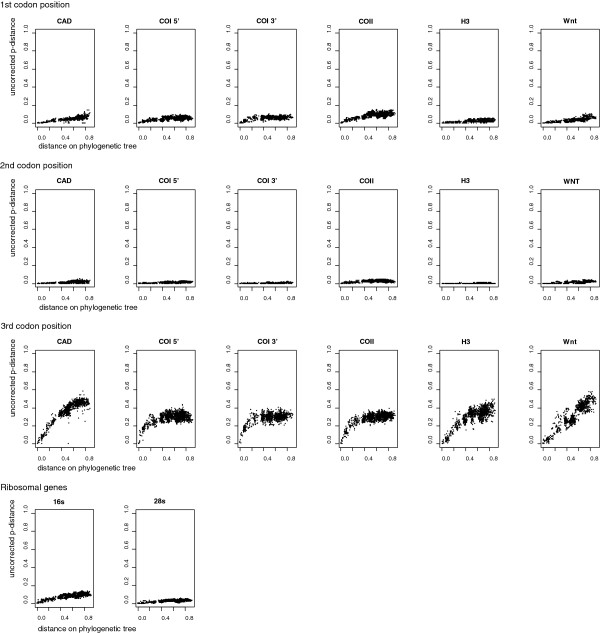
**Saturation plots of genes and codon positions.** Uncorrected p-distances are shown on the y-axis. X-axis represents the pairwise distances as inferred on the tree recovered from the single-gene analysis.

Saturation plots for each gene and codon position (Figure 9) corresponded well with the PI-modified (Figure 8b-c) profile plots. The 3^rd^ codon position of mitochondrial protein-coding genes showed a high level of saturation (Figure 9). In the PI plots (Figure 8a) this can be identified by curves with narrow peaks followed by steep declines. The 3^rd^ codon positions of nuclear protein-coding genes were slightly saturated (CAD, H3) or not saturated at all (Wnt) (Figure 9). No saturation was detected in the 1^st^ and 2^nd^ codons in either mitochondrial or nuclear protein-coding genes, suggesting conservative phylogenetic information in these partitions (Figure 9). In the PI plots this is revealed as low but increasing values backwards in time and without any clear peaks or declines.

## Discussion and conclusion

### Phylogeny

In our analyses, Aciliini was always strongly supported as monophyletic to the exclusion of Eretini (all analyses and partitioning schemes). This is consistent with morphological evidence, in particular the aciliine synapomorphy of having both metatibial spurs apically bifid [12]. The weakly supported alternative of a paraphyletic Aciliini with Eretini nested within [14] can therefore be rejected with the present dataset. Eretini is resolved as the sister clade of Aciliini, also corroborating Miller [12]. This clade (Aciliini + Eretini) is supported by the synapomorphy of having the posterodorsal series of setae on metatibia arranged in a strongly oblique linear series with bases of setae contiguous [12]. The distinctive shape of the larvae is also a likely synapomorphy for Aciliini + Eretini and the two pairs of anterior camera-like stemmata could be a further synapomorphy but needs to be examined for more taxa [16]. The monotypic Eretini was also strongly supported as monophyletic but this has never been questioned since the group is well characterized by several distinctive synapomorphies (see [12,15]).

We recovered a fully resolved backbone and, except in one case, strongly supported relationships between all genera within Aciliini. Three alternative hypotheses had been previously identified, i) sister group relationship between *Acilius* and *Thermonectus*[9,12,13] ii) *Acilius* sister to all other Aciliini [7,11], and iii) *Acilius* and *Graphoderus* as sister taxa [14]. Our results unequivocally support the third hypothesis, i.e. that *Acilius* and *Graphoderus* are sister taxa forming a derived Holarctic clade, corroborating the analyses of Ribera et al. [14]. The *Thermonectus* + *Acilius* hypothesis could be rejected and we found instead the New World *Thermonectus* to be the sister to all other Aciliini. This is a new hypothesis that prompts the investigation of potential morphological synapomorphies for Aciliini excluding the *Thermonectus* clade. A labial palp with 4-6 spines in the larvae could potentially be a synapomorphy but since *Eretes* has four spines it is more likely that the bispined labial palp is a synapomorphy for *Thermonectus*[57]. Since *Thermonectus* is the most species-rich genus its sister-group placement is in fact supported by the quite common pattern that clade diversity and clade ages are positively correlated. It is worth noting that excluding CAD from the dataset resulted in a clade in which *Thermonectus* is sister to Oriental-Australian genera *Rhantaticus* + *Sandracottus*. The sister group relationship between *Rhantaticus* and *Sandracottus* was found in all our analyses as well as in those by Ribera et al. [14]. These are also supported by the synapomorphy of reduced metacoxal lines on the metacoxal process [5,11].

The genus *Tikoloshanes,* includes the single enigmatic and rarely collected species *T. eretiformis*, which is superficially similar to Eretini [5]. Its placement was previously untested. Our conclusion of a monophyletic Aciliini already precludes a close relationship of *Tikoloshanes* with either Eretini or Hydaticini, with which the genus bears some similarity based on the arrangement of the metatibial series of spines, as noted by Omer-Cooper [5]. Despite the mixed characters displayed in *Tikoloshanes*, Omer-Cooper [5] came to the conclusion that *Tikoloshanes* belong to Aciliini with an affinity to the other Afrotropical aciliine genus *Aethionectes*. Our results corroborate this hypothesis, even though it was the only intergeneric node not supported with a posterior probability greater than 0.95 in the four-partition analysis (0.98 in the six partition analysis). Again, the placement changed when CAD was removed resulted in *Tikoloshanes* nested within the Holarctic clade, a very unlikely conclusion.

The effect of removing the 2000 bp CAD fragment should be seen in the light of the data partitions that come to dominate the phylogenetic signal in the new dataset (c.f. Figure 3b). The remaining subset becomes dominated by the mitochondrial genes, since the other nuclear genes are relatively shorter. Most of the phylogenetic signal from the mitochondrial genes is provided by the clearly saturated third codon positions (Figure 9). Placement of a single long branched taxon like *Tikoloshanes* is, likely to be more uncertain when based on a higher proportion of saturated data.

### Biogeography and divergence time

The biogeographical distribution together with the phylogeny of Aciliini and Eretini firmly places the origin of Aciliini in the Southern Hemisphere. The basalmost clades are all optimized to Australia (*Eretes*), South America (*Thermonectus*) and Africa (*Aethionectes* + *Tikoloshanes*). The present distribution could be explained by a vicariance event following the breakup of Gondwana. West Gondwana (South America and Africa) and East Gondwana (India, Madagascar, Australia) was the first major break-up event at about 165-130 Ma [58,59]. The split between ancestral Eretini (optimized to Australian region) and ancestral Aciliini (optimized to S. America and Africa) would follow such a pattern and the age using the fossil calibration on the *Acilius* crown-node was estimated to an interval encompassing that geological age: 95% HPD = 138-88 Ma. Likewise, an early lineage split within Aciliini would have followed the second major break-up in West Gondwana between South America (leading to the divergence of *Thermonectus* in the Neotropical region) and Africa when the South Atlantic ocean started to open at about 135 Ma with land connections likely until around 105 Ma [59]. With the same calibration point this split in the phylogeny was estimated to an interval allowing for a geological vicariance event explanation: (114-74 Ma). At some point the ancestor of the Australasian (*Sandracottus* + *Rhantaticus*) and Holarctic (*Acilius* + *Graphoderus*) clades dispersed out-of-Africa, and this happened long before the Tethys sea actually closed and a land connection was established [60]. Most Aciliini being capable of flight and e.g. *Rhantaticus* having colonized islands like Mauritius and New Caledonia in recent times, this seems reasonable. The out-of-Africa node was dated to 100-65 Ma and during this time Laurasia was divided into an Euramerican and an Asiamerican continent, with a number of smaller continental fragments between these and Africa [61]. The split between the ancestor of Holarctic *Graphoderus* + *Acilius*, optimized to have a Nearctic origin, and the ancestor of Australasian *Rhantaticus* + *Sandracottus*, most probably with a Palearctic/Oriental origin, were dated to 59-90 Ma. The Turgai Strait separated eastern Palearctic from Euramerica during this period [61] and the two clades seem to have their origin on respective sides.

However, estimations using the fossil as an *Acilius* stem-node calibration dated the relevant nodes of the tree to be too young for some of these events. The Aciliini–Eretini split is too young to be explained by the vicariance event of western and eastern Gondwana breaking up (83-57 Ma). The split between Neotropical *Thermonectus* and the rest of Aciliini is too young for the vicariant breakup of South America and Africa (70-49 Ma).

Compared to the crown-group calibration this is a less parsimonious solution, as it requires more transoceanic dispersals events to be invoked. That said, we agree with de Quieroz [62] that we have previously underestimated how geological time scales can make the most improbable dispersal event probable. For increased precision around the evolutionary history of Aciliini, future studies are needed which includes multiple primary calibration points and takes the full uncertainty into account through the priors. Since fossils in this group are scarce this will probably have to be done at higher subfamily, family or superfamily level.

### Phylogenetic informativeness in CAD

In recent years CAD has become commonly used for resolving both shallow and deep evolutionary relationships of insects [19,27,63]. Evaluation of the performance of different gene fragments is often done by comparing single gene tree reconstructions with a total evidence hypothesis or a hypothesis derived from accumulated previous work [27]. But the relative phylogenetic informativeness of genes can also be more directly quantified over time in relation to its evolutionary rate [53,54,64]. While the extrapolation of Townsend’s original formulation of the method [39] beyond the four-taxon case has been questioned [64], modifications have been proposed that seem to more accurately capture the effect of saturation [19]. Our analyses suggested variable phylogenetic informativeness for gene-and codon partitions over the time-scale in this study. CAD contributed substantially to the resolved and highly supported backbone of the phylogenetic tree as evident both from PI plots (Figure 8ab) and from comparing the single gene tree with the total evidence analysis. Analyzed alone CAD recovered both tribes and all genera as monophyletic and resolves the deeper nodes in agreement with a Gondwana breakup influence. This confirms previous studies in Diptera [17] and Hymenoptera [56] where likewise CAD alone successfully resolved Mesozoic-aged divergences in agreement with independent evidence. An evaluation of nuclear genes in Coleoptera [27] also scored CAD as the best performing gene overall among the ones compared, but CAD failed to recover some well-supported Mesozoic-aged clades. This might have had more to do with a poor taxon sampling for this scale and the known effects of taxon sampling [65,66]. For the reconstruction of the Trichoptera tree of life, CAD was surpassed in phylogenetic Informativeness by RNA Polymerase II (POL) at deeper time scales [19].

At 2008 bp, CAD was the longest gene in our dataset, more than three times longer than mitochondrial genes and almost five times longer than other nuclear genes in our dataset. As a consequence, CAD was our most informative gene fragment. To compare its informativeness per site with other genes, we averaged the modified Townsend’s PI profile [19] by dividing PI values with the gene length for each gene and codon position [54]. The Average-modified PI profile indicated that the 3^rd^ codon position of CAD was the most informative between 15 and 50 Ma closely followed and surpassed by the 3^rd^ codon position of Wnt over deeper time. This mirrors the comparison with POL [19], which begs for a future comparison between Wnt and POL over deeper Mesozoic timescales. Moreover, the Average-modified PI plot identified 3^rd^ codon positions of nuclear genes in general as more informative already for relationships older than 15 Ma compared to 3^rd^ codon positions in mitochondrial genes.

It has been reported that CAD 3^rd^ codon position demonstrates heterogeneity in base composition potentially indicating saturation which can mislead phylogenetic analyses [19,37,67]. In our analysis, 3^rd^ codon position of CAD (as well as the 3^rd^ codon positions in Histone 3) was slightly saturated but far less than in mitochondrial genes. Wnt did not show any signs of saturation, even at 3^rd^ codon positions, showing great promise as a marker for deeper relationships but is perhaps underused today due to shortage of primers and protocols to amplify longer fragments. The Wnt gene is also known to produce paralogs for some insect groups [68] and can present alignment problems [27]. The 466 bp target fragment in this study showed no evidence of paralogs, insertions or deletions and was unproblematic to align. The CAD alignment in contrast, displayed a markedly highly variable section located at the 5’ end, including a 3-9 bp long indel, but which translated correctly to amino acids. This variable section, part of the small chain of CPS [17,37], provides CAD with phylogenetic information also at younger levels e.g. within genera.

## Availability of supporting data

The datamatrix for the phylogenetic analysis and Additional file 1: Table S1, Additional file 2: Table S2, Additional file 3: Table S3 are deposited at the DRYAD data depository (DOI:10.5061/dryad.052ng).

## Competing interests

The authors declare that they have no competing interests.

## Authors' contributions

JB and RB conceived the study. RB and KBM performed the lab work. JB and KBM provided crucial samples. RB and JB performed the analysis and wrote the first draft. All authors contributed to the final manuscript. All authors read and approved the final manuscript.

## Supplementary Material

Additional file 1: Table S1 Specimen data and NCBI accession numbers. Click here for file

Additional file 2: Table S2 Primers. Primers used for amplification and sequencing were derived from several sources. Click here for file

Additional file 3: Table S3 Summary of the partitions and models. The best-fit partitioning scheme and model according to PartitionFinder. Click here for file

## References

[B1] Nilsson AN Dytiscidae World Catalogue of Insects: 1-395 2001

[B2] Larson DJ Alarie Y RRE: Predaceous Diving Beetles (Coleoptera: Dytiscidae) of the Nearctic Region, with Emphasis on the Fauna of Canada and Alaska 2000 Ottawa: NRC Research Press

[B3] Bergsten J Miller KB Taxonomic revision of the Holarctic diving beetle genus Acilius Leach (Coleoptera: Dytiscidae) Syst Entomol 2005 31 145 197 10.1111/j.1365-3113.2005.00309.x

[B4] Nilsson AN Holmen M The aquatic Adephaga (Coleoptera) of Fennoscandia and Denmark: II: dytiscidae Fauna Entomol Scand 1995 32 192pp

[B5] Omer-Cooper J Tikoloshanes, a new genus of Dytiscidae (Col.) from South Africa Proc R Entomol Soc London 1956 79 82

[B6] Bergsten J Miller KB Phylogeny of diving beetles reveals a coevolutionary arms race between the sexes PloS one 2007 2 e522 10.1371/journal.pone.0000522 17565375 PMC1885976

[B7] Erichson WF Genera Dyticeorum 1832 Berolini: Nietackianis 48

[B8] Dejean PFMA Catalogue Des Coléoptères De La Collection De M. Le Comte Dejean 1833 Paris: Livraisons 1 & 2 176

[B9] Aubé C Dejean PF Hydrocanthares Et Gyriniens Species Général Des Coléoptères De La Collection De M. Le Comte Dejean 1838 6 Paris: Méquignon Père et Fils 804

[B10] Régimbart M Étude Sur La Classification Des Dytiscidae Annales de la Société Entomologique de France (5) 1879 8 1878 447 466 +?pl. 10:1-28

[B11] Sharp D On aquatic carnivorous coleoptera or dytiscidae Sci Trans R Dublin Soc 1882 2 2 179 1003 +psl. 7-18

[B12] Miller KB Revision of the Neotropical Genus Hemibidessus Zimmermann (Coleoptera: Dytiscidae: Hydroporinae: Bidessini) Aquat Insects 2000 23 253 275

[B13] Miller KB On the phylogeny of the Dytiscidae (Insecta: Coleoptera) with emphasis on the morphology of the female reproductive system Insect Syst Evol 2001 32 45 92 10.1163/187631201X00029

[B14] Ribera I Vogler AP Balke M Phylogeny and diversification of diving beetles (Coleoptera: Dytiscidae) Cladistics 2008 24 563 590 10.1111/j.1096-0031.2007.00192.x 34879635

[B15] Miller KB Revision of the Genus Eretes Laporte, 1833 (Coleoptera: Dytiscidae) Aquat Insects 2002 24 247 272 10.1076/aqin.24.4.247.8238

[B16] Mandapaka K Morgan RC Buschbeck EK Twenty-eight retinas but only twelve eyes: an anatomical analysis of the larval visual system of the diving beetle Thermonectus marmoratus (Coleoptera: Dytiscidae) J Comp Neurol 2006 497 166 181 10.1002/cne.20974 16705677

[B17] Moulton JK Wiegmann BM Evolution and phylogenetic utility of CAD (rudimentary) among Mesozoic-aged Eremoneuran Diptera (Insecta) Mol Phylogenet Evol 2004 31 363 378 10.1016/S1055-7903(03)00284-7 15019631

[B18] Richards S Gibbs RA Weinstock GM Brown SJ Denell R Beeman RW Gibbs R Bucher G Friedrich M Grimmelikhuijzen CJP Klingler M Lorenzen M Roth S Schröder R Tautz D Zdobnov EM Muzny D Attaway T Bell S Buhay CJ Chandrabose MN Chavez D Clerk-Blankenburg KP Cree A Dao M Davis C Chacko J Dinh H Dugan-Rocha S Fowler G The genome of the model beetle and pest Tribolium castaneum Nature 2008 452 949 955 10.1038/nature06784 18362917

[B19] Malm T Johanson K Wahlberg N The evolutionary history of Trichoptera (Insecta): a case of successful adaptation to life in freshwater Syst Entomol 2013 38 459 473 10.1111/syen.12016

[B20] Danforth BN Fang J Sipes S Analysis of family-level relationships in bees (Hymenoptera: Apiformes) using 28S and two previously unexplored nuclear genes: CAD and RNA polymerase II Mol Phylogenet Evol 2006 39 358 372 10.1016/j.ympev.2005.09.022 16412668

[B21] Praz CJ Müller A Danforth BN Griswold TL Widmer A Dorn S Phylogeny and biogeography of bees of the tribe Osmiini (Hymenoptera: Megachilidae) Mol Phylogenet Evol 2008 49 185 197 10.1016/j.ympev.2008.07.005 18675365

[B22] Winterton SL Hardy NB Wiegman BM On wings of lace: phylogeny and Bayesian divergence time estimates of Neuropterida (Insecta) based on morphological and molecular data Syst Entomol 2010 35 349 378 10.1111/j.1365-3113.2010.00521.x

[B23] Johanson KA Malm T Testing the monophyly of Calocidae (Insecta: Trichoptera) based on multiple molecular data Mol Phylogenet Evol 2010 54 535 541 10.1016/j.ympev.2009.09.025 19786110

[B24] Regier JC Zwick A Cummings MP Kawahara AY Cho S Weller S Roe A Baixeras J Brown JW Parr C Davis DR Epstein M Hallwachs W Hausmann A Janzen DH Kitching IJ Solis MA Yen S-H Bazinet AL Mitter C Toward reconstructing the evolution of advanced moths and butterflies (Lepidoptera: Ditrysia): an initial molecular study BMC Evol Biol 2009 9 280 10.1186/1471-2148-9-280 19954545 PMC2796670

[B25] Dole SA Jordal BH Cognato AI Polyphyly of xylosandrus reitter inferred from nuclear and mitochondrial genes (Coleoptera: Curculionidae: Scolytinae) Mol Phylogenet Evol 2010 54 773 782 10.1016/j.ympev.2009.11.011 19925873

[B26] Jordal BH Sequeira AS Cognato AI The age and phylogeny of wood boring weevils and the origin of subsociality Mol Phylogenet Evol 2011 59 708 724 10.1016/j.ympev.2011.03.016 21435394

[B27] Wild AL Maddison DR Evaluating nuclear protein-coding genes for phylogenetic utility in beetles Mol Phylogenet Evol 2008 48 877 891 10.1016/j.ympev.2008.05.023 18644735

[B28] Maddison DR Arnold EA A review of the Bembidion (Odontium) aenulum subgroup (Coleoptera: Carabidae), with description of a new species ZOOTAXA 2009 2214 45 61

[B29] Mousseau T Sikes DS Almost but not quite a subspecies: a case of genetic but not morphological diagnosability in Nicrophorus (Coleoptera: Silphidae) Biol J Linn Soc 2011 102 311 333 10.1111/j.1095-8312.2010.01568.x

[B30] Kaltenpoth M Steiger S Unearthing carrion beetles’ microbiome: characterization of bacterial and fungal hindgut communities across the Silphidae Mol Ecol 2013 doi:10.1111/mec.12469 10.1111/mec.12469 24102980

[B31] Sikes DS Venables C Molecular phylogeny of the burying beetles (Coleoptera: Silphidae: Nicrophorinae) Mol Phylogenet Evol 2013 69 552 565 10.1016/j.ympev.2013.07.022 23911726

[B32] Maddison DR Systematics of the North American beetle subgenus Pseudoperyphus (Coleoptera: Carabidae: Bembidion) based upon morphological, chromosomal, and molecular data Ann Carnegie Mus 2008 77 147 193 10.2992/0097-4463-77.1.147

[B33] Maddison DR Toledano L Sallenave S Roig-Junent S Phylogenetic relationships of the South American ground beetle subgenus Chilioperyphus Jeannel (Coleoptera: Carabidae: Trechinae: Bembidiini: Bembidion Latreille) ZOOTAXA 2013 3636 547 560 10.11646/zootaxa.3636.4.3 26042310

[B34] Maddison DR Phylogeny of Bembidion and related ground beetles (Coleoptera: Carabidae: Trechinae: Bembidiini: Bembidiina) Mol Phylogenet Evol 2012 63 533 576 10.1016/j.ympev.2012.01.015 22421212

[B35] Sharanowski BJ Dowling APG Sharkey MJ Molecular phylogenetics of Braconidae (Hymenoptera: Ichneumonoidea), based on multiple nuclear genes, and implications for classification Syst Entomol 2011 36 549 572 10.1111/j.1365-3113.2011.00580.x

[B36] Miller KB Bergsten J Whiting MF Phylogeny and classification of the tribe Hydaticini (Coleoptera: Dytiscidae): partition choice for Bayesian analysis with multiple nuclear and mitochondrial protein-coding genes Zool Scr 2009 38 591 615 10.1111/j.1463-6409.2009.00393.x

[B37] Miller KB Bergsten J Phylogeny and classification of whirligig beetles (Coleoptera: Gyrinidae): relaxed-clock model outperforms parsimony and time-free Bayesian analyses Syst Entomol 2012 37 706 746 10.1111/j.1365-3113.2012.00640.x

[B38] Edgar RC MUSCLE: multiple sequence alignment with high accuracy and high throughput Nucleic Acids Res 2004 32 1792 1797 10.1093/nar/gkh340 15034147 PMC390337

[B39] Tamura K Peterson D Peterson N Stecher G Nei M Kumar S MEGA5: molecular evolutionary genetics analysis using maximum likelihood, evolutionary distance, and maximum parsimony methods Mol Biol Evol 2011 28 2731 2739 10.1093/molbev/msr121 21546353 PMC3203626

[B40] Peña C Malm T VoSeq: a voucher and DNA sequence web application PloS one 2012 7 e39071 10.1371/journal.pone.0039071 22720030 PMC3373637

[B41] Lanfear R Calcott B Ho SYW Guindon S Partitionfinder: combined selection of partitioning schemes and substitution models for phylogenetic analyses Mol Biol Evol 2012 29 1695 1701 10.1093/molbev/mss020 22319168

[B42] Ronquist F Teslenko M Van der Mark P Ayres DL Darling A Höhna S Larget B Liu L Suchard MA Huelsenbeck JP MrBayes 3.2: efficient Bayesian phylogenetic inference and model choice across a large model space Syst Biol 2012 61 539 542 10.1093/sysbio/sys029 22357727 PMC3329765

[B43] Huelsenbeck JP Larget B Alfaro ME Bayesian phylogenetic model selection using reversible jump Markov chain Monte Carlo Mol Biol Evol 2004 21 1123 1133 10.1093/molbev/msh123 15034130

[B44] Drummond AJ Rambaut A BEAST: Bayesian evolutionary analysis by sampling trees BMC Evol Biol 2007 7 214 10.1186/1471-2148-7-214 17996036 PMC2247476

[B45] Wilgenbusch JC Warren DL Swofford DL AWTY: a system for graphical exploration of MCMC convergence in Bayesian phylogenetic inference 2004 http://ceb.csit.fsu.edu/awty 10.1093/bioinformatics/btm388 17766271

[B46] Nylander JAA Wilgenbusch JC Warren DL Swofford DL AWTY (are we there yet?): a system for graphical exploration of MCMC convergence in Bayesian phylogenetics Bioinformatics (Oxford, England) 2008 24 581 583 10.1093/bioinformatics/btm388 17766271

[B47] Drummond AJ Ho SYW Phillips MJ Rambaut A Relaxed phylogenetics and dating with confidence PLoS biology 2006 4 e88 10.1371/journal.pbio.0040088 16683862 PMC1395354

[B48] Evanoff E Mcintosh WC MPC Evanoff E, Gregory Wodzicki KM, Johnson KR Stratigraphic summary and 40Ar/39Ar. geochronology of the Florissant Formation, Colorado Proceedings of the Denver Museum of Nature and Science 2001 4 1 1 16

[B49] Wickham HF New fossil Coleoptera from Florissant Am J Sci Arts 1909 4 126 130

[B50] Ho SYM Calibrating molecular estimates of substitution rates and divergence times in birds J Avian Biol 2007 38 409 414

[B51] Ho SYW Phillips MJ Accounting for calibration uncertainty in phylogenetic estimation of evolutionary divergence times Syst Biol 2009 58 367 380 10.1093/sysbio/syp035 20525591

[B52] Yu Y Harris AJ He XJ RASP (Reconstruct Ancestral State in Phylogenies) 2.1 beta 2013 http://mnh.scu.edu.cn/soft/blog/RASP 10.1016/j.ympev.2015.03.008 25819445

[B53] Townsend JP Profiling phylogenetic informativeness Syst Biol 2007 56 222 231 10.1080/10635150701311362 17464879

[B54] Townsend JP Su Z Tekle YI Phylogenetic signal and noise: predicting the power of a data set to resolve phylogeny Syst Biol 2012 61 835 849 10.1093/sysbio/sys036 22389443

[B55] Klopfstein S Vilhelmsen L Heraty JM Sharkey M Ronquist F The hymenopteran tree of life: evidence from protein-coding genes and objectively aligned ribosomal data PLoS ONE 2013 8 e69344 10.1371/journal.pone.0069344 23936325 PMC3732274

[B56] Pond SLK Frost SDW Muse SV HyPhy: hypothesis testing using phylogenies Bioinformatics (Oxford, England) 2005 21 676 679 10.1093/bioinformatics/bti079 15509596

[B57] Bertrand H Larves Et Nymphes Des Coléoptères Aqua- Tiques Du Globe 1972 France: F. Paillart 804

[B58] Yoder AD Nowak MD Has vicariance or dispersal been the predominant biogeographic force in Madagascar? Only time will tell Annu Rev Ecol Evol Syst 2006 37 405 431 10.1146/annurev.ecolsys.37.091305.110239

[B59] Stephen M The breakup history of Gondwana and its impact on pre-Cenozoic floristic provincialism Aust J Bot 2001 49 271 300 10.1071/BT00023

[B60] Hrbek T Meyer A Closing of the Tethys Sea and the phylogeny of Eurasian killifishes (Cyprinodontiformes: Cyprinodontidae) J Evol Biol 2003 16 17 36 10.1046/j.1420-9101.2003.00475.x 14635877

[B61] Sanmartin I Enghoff H Ronquist F Patterns of animal dispersal, vicariance and diversification in the Holarctic Biol J Linn Soc 2001 73 345 390

[B62] De Queiroz A The resurrection of oceanic dispersal in historical biogeography Trends Eco Evol 2005 20 68 73 10.1016/j.tree.2004.11.006 16701345

[B63] Wiegmann BM Trautwein MD Kim J-W Cassel BK Bertone MA Winterton SL Yeates DK Single-copy nuclear genes resolve the phylogeny of the holometabolous insects BMC Biol 2009 7 34 10.1186/1741-7007-7-34 19552814 PMC2709105

[B64] Klopfstein S Kropf C Quicke DLJ An evaluation of phylogenetic informativeness profiles and the molecular phylogeny of diplazontinae (Hymenoptera, Ichneumonidae) Syst Biol 2010 59 226 241 10.1093/sysbio/syp105 20525632

[B65] Zwickl DJ Hillis DM Increased taxon sampling greatly reduces phylogenetic error Syst Biol 2002 51 588 598 10.1080/10635150290102339 12228001

[B66] Heath TA Hedtke SM Hillis DM Taxon sampling and the accuracy of phylogenetic analyses J Syst Evol 2008 46 239 257

[B67] Xia X Xie Z Salemi M Chen L Wang Y An index of substitution saturation and its application Mol Phylogenet Evol 2003 26 1 7 10.1016/S1055-7903(02)00326-3 12470932

[B68] Danforth BN Brady SG Sipes SD Pearson A Single-copy nuclear genes recover cretaceous-age divergences in bees Syst Biol 2004 53 309 326 10.1080/10635150490423737 15205055

