# Supplementary material for: The utility of CAD in recovering Gondwanan vicariance events and the evolutionary history of Aciliini (Coleoptera: Dytiscidae)
Source: BMC Evol Biol. 2014 Jan 14;14:5. doi: 10.1186/1471-2148-14-5 (PMC3901756; doi:10.1186/1471-2148-14-5)
Supplement: Additional file 1: Table S1 — Specimen data and NCBI accession numbers. [file 1471-2148-14-5-S1.pdf]

Genus	Species	Collecting data	Code	COI_3'	COI_5'	COII	16S	H3	WNT	CAD	28S
Eretes	australis	Australia: 15km N Kingston, 34º 5.833' S, 140º 21' E, 12:XI:2000, Leg. K. Miller	KBMErau103	FJ796579	---	KF978919	KF979073	FJ796506	FJ796547	---	---
Eretes	explicitus	USA, Texas, Bailey Co. Muleshoe National Wildlife Refuge, 30:VII:2003, Leg. O. Bocanegra	MNCN-AI1257	KF979041	---	----	----	---	---	---	----
Eretes	griseus	Zambia, 50km E. Isoka, 4:XII:2004, Leg. Werner, Smrz	412C12 BMNH832133	KF979043	---	KF978920	KF979074	KF978969	---	KF978871	KF978819
Eretes	griseus	Madagascar, Toliara, 80 km E Toliara, RN10, 23º 14.54' S, 44º 13.75' E, 416m, 17:V:2006, Leg. J. Bergsten	164G05 BMNH742611	KF979042	---	KF978921	KF979075	KF978970	---	KF978872	KF978820
Eretes	sticticus	South Africa, Eastern Cape Province, 2km N. Sterkstroom, 31º 30.233' S, 26º 32.16', 1414m, 20-28:I:2005, Leg. J. Bergsten	411G2 BMNH832075	KF979045	---	KF978922	KF979076	---	---	KF978873	KF978821
Eretes	sticticus	Madagascar, Toliara, Arbor d'Antsokay, 10:XI:2005, Leg. J. Stastný	133A06 BMNH729837	KF979044	---	KF978923	KF979077	KF978971	---	KF978874	KF978822
Acilius	abbreviatus	USA, Idaho, Fremont Co., 44º 30.372' N, 111º 16.56' W,1957m, 16:IX:2000, Leg. Gustafson	KBMAcab72	KF979029	KF979029	KF978924	KF979078	KF978972	KF979001	KF978875	KF978823
Acilius	athabascae	Canada, Alberta: 8km SE of Bearberry, 8:IX:2002, Leg. J. Bergsten	305G09 BMNH799009	KF979030	KF979030	KF978925	KF979079	KF978973	---	KF978876	KF978824
Acilius	canaliculatus	Sweden, Vb, Umeå: Nydalasjön, 19:V:2000, J. Bergsten	KBMAcca70	DQ275298	KF979031	KF978926	KF979080	DQ275314	DQ275330	KF978877	KF978825
Acilius	confusus	USA, Maryland, Talbot, 3km SE Easton, 3:VIII:1986, Leg. W. Steiner, J. Hill, J. Swearingen	KBMAcco150	KF979032	KF979032	KF978927	KF979081	---	---	---	KF978826
Acilius	confusus	USA, Maryland, Talbot, 3km SE Easton, 3:VIII:1986, Leg. W. Steiner, J. Hill, J. Swearingen	KBMAcco157	DQ275299	---	---	---	DQ275315	DQ275331	---	---
Acilius	duvergeri	Italy, Sardinia, Giara de Gesturi, Funtana s'Ala de Mengiaru, 21:VI:2000, Leg. J. Bergsten	KBMcdu50	DQ275300	KF979033	KF978928	---	DQ275316	DQ275328	KF978878	KF978827
Acilius	duvergeri	Portugal	BMNH681349	---	---	---	AJ850357	---	---	---	---
Acilius	fraternus	USA,New York, Beard Mtn., Over Look Lodge,13:VII:2000, Leg, C. Hernando	KBMAcfr115	DQ275301	KF979034	KF978929	---	DQ275317	DQ275329	KF978879	KF978828
Acilius	japonicus	Japan, Honshu,Yamagata Pref., Shizu, Nishikawa, 12:VIII:2000, Leg, N. Hikida	KBMAcja84	DQ275302	KF979035	KF978930	KF979082	DQ275320	DQ275326	KF978880	KF978829
Acilius	kishii	Japan, Honshu, Fukui Pref. Yashaga-Ike, 30:VIII:2000, Leg, J. Bergsten	KBMAcki83	DQ275305	KF979036	KF978931	KF979083	DQ275321	DQ275327	KF978881	KF978830
Acilius	mediatus	USA, New York, Tompkins Co., pond 10km S Caroline, 2:IX:2000, Leg. K. Miller	KBMAcme6	DQ275306	KF979037	KF978932	KF979084	DQ275322	AF391998	KF978882	KF978831
Acilius	semisulcatus	USA, New York, Tompkins Co., pond 10km S Caroline, 2:IX:2000, Leg. K. Miller	KBMAcse7	DQ275307	---	KF978933	KF979085	DQ275323	AF391999	KF978883	KF978832
Acilius	sulcatus	Russia, Sakhalin, SK-01-BM-026	KBMAcsu155	---	KF979038	KF978934	---	---	---	---	---
Acilius	sulcatus	Sweden, Umeå, Lomtjärn, 22:VI:2000, Leg. J. Bergsten	KBMAcsu49	DQ275308	---	---	---	DQ275324	AF392000	---	---

Acilius	sylvanus	USA, New York: St Lawrence Co., Fish Cr Marsh, 44º 28.333' N, 75º 33.8' W, 12:XI:2000, Leg. K.Miller	KBMAcsy51	DQ275309	KF979039	KF978935	KF979086	DQ275325	AF392001	KF978884	KF978833
Aethionectes	oberthueri	Madagascar: Andasibe National Park, 18º 56.19' S, 48º 25.18' E, 950m, 1:IV:2007, Leg.	393H5 BMNH829993	HQ383199	---	KF978936	KF979087	KF978974	KF979002	KF978885	KF978834
Aethionectes	apicalis	South Africa, Eastern Cape Province, Dwesa NP,	KBMAeop406	KF979040	KF979040	KF978937	KF979088	KF978975	---	KF978886	KF978835
Graphoderus	adamsii	Japan, Hokkaido, Nopporo Forest Park, 23:VIII:2000, Leg. J.Bergsten	KBMGrad188	KF979046	KF979046	KF978938	KF979089	KF978976	KF979003	KF978887	KF978836
Graphoderus	austriacus	Russia, Volgograd Obl., Volgograd, Kiriova, 9:V:2000, Leg. J. Bergsten	KBMGrau190	KF979047	KF979047	KF978939	KF979090	KF978977	KF979004	KF978888	KF978837
Graphoderus	bieneri	Russia, Pimorye, Sputnik Station/P7, 28:VII:1993 Leg. S. Khalin	KBMGrbe246	KF979048	KF979048	KF978940	KF979091	KF978978	KF979005	KF978889	KF978838
Graphoderus	bilineatus	Russia, Volgograd Obl., Krasnoslobodsk, 15:V:2001, Leg. J.Bergsten	KBMGrbi193	KF979049	KF979049	KF978941	KF979092	KF978979	KF979006	KF978890	KF978839
Graphoderus	cinereus	Russia, Volgograd Obl., Volgograd, Kiriova, 9:V:2000, Leg. J. Bergsten	KBMGrci198	KF979050	KF979050	KF978942	---	KF978980	KF979007	KF978891	KF978840
Graphoderus	cinereus	Spain, Leg. I. Ribera		---	---	---	AY138647	---	---	---	---
Graphoderus	fascicollis	USA, New York, Oswego Co., nr Boylstown Center, 43º 39.433' N, 25º 55.117' W, 18:VIII:2000, Leg. K.Miller	KBMGrfa65	DQ275310	KF979051	KF978943	KF979093	DQ275318	AF392015	KF978892	KF978841
Graphoderus	liberus	USA, New York, Tompkins Co., Ringwood Preserve, 19:IX:2000, Leg. K. Miller	KBMGrli69	DQ813693	DQ813693	DQ813795	KF979094	DQ813759	AF392016	KF978893	KF978842
Graphoderus	occidentalis	Canada, Alberta W4merTwp68 Rge24 Sec10SE, 2:IX:2002, Leg. J. Bergsten	KBMGroc207	KF979052	KF979052	KF978944	KF979095	KF978981	KF979008	KF978894	KF978843
Graphoderus	perplexus	Canada, Alberta, W4mer Twp77 Rge14, 4:IX:2002, Leg. J. Bergsten	KBMGrpe200	JF499705	KF979053	KF978945	KF979096	KF978982	KF979009	KF978895	KF978844
Graphoderus	zonatus	Sweden, Umeå, Lomtjärn, 22:VI:2000, Leg. J. Bergsten	KBMGrzo66	DQ275311	KF979054	KF978946	KF979097	DQ275319	AF392017	KF978896	KF978845
Rhantaticus	congestus	Australia, NT, Litchfield NP, 223m, 13.10.489S 130.44.575E, 21:VIII:2006, Leg. L & H hendrich	MB1684	KF979025	KF979025	KF978947	KF979098	---	KF979010	KF978897	KF978846
Rhantaticus	congestus	New Caledonia, Leg. Damgaard	MB3849	KF979026	KF979026	KF978948	KF979099	---	KF979011	KF978898	KF978847
Rhantaticus	congestus	Zambia, 50km E. Isoka, 9:XII:2004, Leg. Werner, Smrz	412B12 BMNH832121	---	---	KF978949	KF979100	KF978983	---	KF978899	KF978848
Rhantaticus	congestus	Nepal, Guanganagar, Chitwom, V:2005, Leg. D. Ahrens	412C2 BMNH832123	---	KF979056	KF978950	KF979101	KF978984	---	KF978900	KF978849
Rhantaticus	congestus	Madagascar, Toliara, Kirindy reserve, 20.07476 S, 44.67075 E, 12:XII:2009, Leg. J. Bergsten	NHRS-JLKB000000097	KF979058	KF979058	KF978951	KF979102	---	KF979012	KF978901	KF978850
Rhantaticus	congestus	Mauritius, Rodrigues, Mont Kall, 17:XII:2005, Leg. C. Turner	NHRS-JLKB000000091	KF979057	KF979057	KF978952	KF979103	KF978985	KF979013	KF978902	KF978851
Sandracottus	bakewelii	Australia, NT, Ormiston, Leg. C. Watts	KBMSaba90	KF979059	KF979059	KF978953	KF979104	KF978986	KF979014	KF978903	KF978852

Sandracottus	dejeanii	Nepal, Guanganagar, Chitwom, V:2005, Leg. D. Ahrens	412D3 BMNH832136	KF979060	---	---	---	KF978987	---	KF978904	KF978853
Sandracottus	dejeanii	India, Maharastra, 16º 34.992' N 73º 35.221' E, 1:X:2004, Leg. K. Miller	KBMSade328	---	---	KF978954	---	---	---	---	---
Sandracottus	fasciatus	Thailand, Phitsanoluk Chat Trakan, 10:I:2009, Leg. H. Freitag	MB3686	KF979027	KF979027	KF978955	KF979105	KF978988	KF979015	KF978905	KF978854
Sandracottus	guerini	P.N.Guinea, East Sepik, Lembena, 04º 57.512'S, 143º 57.366' E, 10:IX:2009, Leg. Ibalim, Pius	NHRS-RASA000000015	KF979061	KF979061	---	---	KF978989	---	---	---
Sandracottus	insignis	Phillippines, N. Luzon, Pampanga, Bano San Juan, Mt. Arayat NP, 15°10'21''N, 120°45'22''E, 31:X:2009, Leg. H. Freitag	MB4015	KF979028	KF979028	KF978956	KF979106	KF978990	KF979016	KF978906	KF978855
Thermonectus	alfredi	Bolivia, Dpto. Santa Cruz, Prov. Florida, Rio del Sauce, 1.3km NE Samaipata, 30:VI:1999, Leg. K.Miller	KBMThal264	KF979062	---	KF978957	KF979107	---	---	---	KF978856
Thermonectus	basillaris	USA, New York, Schuyler Co., Texas Hollow, 42º 24.73' N 76º 47.58' W, 358m, 27:Vi:1999, Leg. K.Miller	KBMThba210	DQ431223	KF979063	KF978958	KF979108	KF978991	KF979017	KF978907	KF978857
Thermonectus	circumscriptus	Bolivia, Dpto. Santa Cruz, Prov. Chiquitos, 2.7km S San Jose, 17º 52.333' S 60º 44.467' W, 27:VI:1999, Leg. K. Miller	KBMThci45	---	---	KF978959	KF979109	KF978992	KF979018	KF978908	KF978858
Thermonectus	circumscriptus	Peru, Madre de Dios, 20km S Infierno nr Puerto Maldonado, 14:XII:2003, Leg. K. Miller	KBMThci257	DQ431224	---	---	---	---	---	---	---
Thermonectus	intermedius	USA, California: Yolo CO. rd 27 about 4km N Davis, 28:IX:2002; Leg. J. Bergsten	KBMThin208	KF979064	KF979064	KF978960	KF979110	KF978993	KF979019	KF978909	KF978859
Thermonectus	leprieuri	Peru, Madre de Dios: Explorers Inn, 12º 50.208' S 69º 17.603' W, 10:XII:2003, Leg. K. Miller	KBMThle259	DQ431225	KF979065	KF978961	KF979111	KF978994	KF979020	KF978910	KF978860
Thermonectus	marmoratus	USA, Arizona, Pima Co., 6km S Arivaca, 31º 31.25' N 111º 16.683' W, 1088m, 29:III:2000, Leg. K. Miller	KBMThma13	DQ431227	KF979067	KF978962	KF979112	---	AF392046	KF978911	KF978861
Thermonectus	nigrofasciatus	USA, Arizona, Cochise Co., Rucker Canyon, 31º 45.067' N 109º 22' W, 26:III:2000, Leg. K.Miller	KBMThni5	KF979068	KF979068	KF978963	KF979113	KF978995	AF392047	KF978912	KF978862
Thermonectus	nobilis	Peru, Madre de Dios: boat landing nr Infierno, 12º 43.49333' S 69º 13.04333' W, 14:XII:2003, Leg. K. Miller	KBMThno256	DQ431228	KF979069	KF978964	KF979114	KF978996	KF979021	---	KF978863
Thermonectus	succinctus	Bolivia, Dpto. Santa Cruz Prov. Chiquitos, 2.2km E San Jose, 17º 51' S 60º 43.2' W, 26:VI:1999, Leg. K. Miller	KBMThsu265	DQ431229	KF979070	KF978965	KF979115			KF978913	KF978864
Thermonectus	succinctus	Bolivia, Dpto. Santa Cruz, Prov. Chiquitos, 20km W San Jose, 17º 44.903' S 61º 4.202' W, 13:I:2004, Leg. Svenson	KBMThsu267	---	---	---	---	KF978997	KF979022	---	---

Thermonectus	variegatus	Peru, Madre de Dios: Explorers Inn, 12º 50.208' S 69º 17.603' W, 10:XII:2003, Leg. K. Miller	KBMThva258	DQ431231	KF979071	KF978966	KF979116	KF978998	KF979023	KF978914	KF978865
Thermonectus	margineguttatus	Costa Rica, Alajuela, CR 5, Cano Negro area, 50m, 12:VIII:2002, Leg. M. Balke	KBMThmg199	KF979066	KF979066	KF978967	KF979117	KF978999	KF979024	KF978915	KF978866
Tikoloshanes	eretiformis	South Africa, Eastern Cape Province, 2km N Sterkstroom, 31º 30.233' S, 26º 32.16', 1414m, 20:I:2005, Leg. J. Bergsten	KBMTier407	KF979072	KF979072	KF978968	KF979118	KF979000	---	KF978916	KF978867
Hydaticus	aruspex	France, Gironde, St. Magne, 8:V:2010, Leg. F. Bameul	NHRS-JLKB000000298	KF979055	---	---	KF979119	---	---	KF978917	KF978868
Hydaticus	aruspex	USA, New York, Schuyler Co. Texas Hollow, 6:IX:2000, Leg. K. Miller	KBMHyar68	---	FJ796580	FJ796627	---	FJ796507	AF392019	---	---
Hydaticus	transversalis	France, Isère, La Verpilliere, 13:VII:2005, Leg. G. Foster	127A06 BMNH726388	---	---	---	---	---	---	---	KF978869
Hydaticus	transversalis	Russia, Volgograd Obl., Krasnoslobodsk, 15:V:2001, Leg. J.Bergsten	KBMHytr192	FJ796619	FJ796619	FJ796663	---	FJ796540	FJ796575	---	---
Hydaticus	transversalis	UK, Sommerset Levels, Catcott Heath, 4:VII:1998, I. Ribera	BMNH681265	---	---	---	AJ850365	---	---	---	---
Hydaticus	leander	Portugal, Odemira: Vila Nova de Milfontes, Planalto do Malhao, 6:VI:2000, Leg. J. Bergsten	KBMCHyle195	---	FJ796598	FJ796645	AJ850364	FJ796523	FJ796558	---	---
Dytiscus	verticalis	USA, New York, Tompkins Co., Ithaca, 26:V:2000, Leg. K. Miller	KBMCDyve24	---	DQ813692	DQ813794	---	DQ813758	AF392012	---	---
Dytiscus	verticalis	USA, Maryland: Easton: Seth State Forest, 30:VII:2003, Leg. J.Bergsten	315B02 BMNH799902	---	---	---	KF979120	---	---	KF978918	KF978870
