# Supplementary material for: The utility of CAD in recovering Gondwanan vicariance events and the evolutionary history of Aciliini (Coleoptera: Dytiscidae)
Source: BMC Evol Biol. 2014 Jan 14;14:5. doi: 10.1186/1471-2148-14-5 (PMC3901756; doi:10.1186/1471-2148-14-5)
Supplement: Additional file 2: Table S2 — Primers. Primers used for amplification and sequencing were derived from several sources. [file 1471-2148-14-5-S2.pdf]

Table 2. **Primers.** Primers used for amplification and sequencing were derived from several sources.

Gene	Primers	Sequence	Source
CAD	CD439F	TTC AGT GTA CAR TTY CAY CCH GAR CAY AC	[31]
CAD	CD688R	TGT ATA CCT AGA GGA TCD ACR TTY TCC ATR TTR CA	[31]
CAD	CD667F	GGA TGG AAG GAA GTD GAR TAY GAR GT	[31]
CAD	CD821F	AGC ACG AAA ATH GGN AGY TCN ATG AAR AG	[31]
CAD	CD851R	GGA TCG AAG CCA TTH ACA TTY TCR TCH ACC AT	[31]
CAD	CD1098R2	GCT ATG TTG TTN GGN AGY TGD CCN CCC AT	[31]
COII	F-lue2	TCT AAT ATG GCA GAT TAG TGC	[74]
COII	R-lys2	GAG ACC AGT ACT TGC TTT CAG TCA TC	[74]
H3	Haf3	ATG GCT CGT ACC AAG CAG ACG GC	[75]
H3	Har3	ATA TCC TTG GGC ATG ATG GTG AC	[75]
COI	HCO	TAAACTTCAGGGTGACCAAAAAATCA	[76]
COI	LCO	GGTCAACAAATCATAAAGATATTGG	[76]
COI	Jerry	CAA CAT TTA TTT TGA TTT TTT GG	[74]
COI	PatDyt	TCA TTG CAC TAA TCT GCC ATA TTAC	[77]
COI	COI_RB_r2	ACTAATGGAAATGAGCAACAACA	this study
COI	COI_RB_f2	GAGCTTATTTTACTTCAGCAACT	this study
Wnt	Wnt_RB_F1	TGCGTCTTCCATCATTCCGT	this study
Wnt	WNT_RB_R1	CGATATCCTCTACCACAACACATTA	this study
Wnt	LepWg1	GAR TGY AAR TGY CAY GGY ATG TCT GG	[78]
Wnt	LepWg2a4	ACT ICG CAR CAC CAR TGG AAT	[78]
Wnt	WgDytF1	CGY CTT CCW TCW TTC CGW GTY ATC	[79]
Wnt	WgDytR1	CCG TGG ATR CTG TTV GCH AGA TG	[79]
16S	16Sar-fw	CGCCTGTTTAACAAAAACAT	[80]
16S	16Sb2-rw	TTTAATCCAACATCGAGG	[80]
28S	28S-DD	GGGACCCGTCTTGAAACAC	[81]
28S	28S-FF	TTACACACTCCTTAGCGGAT	[81]
