# Supplementary material for: The utility of CAD in recovering Gondwanan vicariance events and the evolutionary history of Aciliini (Coleoptera: Dytiscidae)
Source: BMC Evol Biol. 2014 Jan 14;14:5. doi: 10.1186/1471-2148-14-5 (PMC3901756; doi:10.1186/1471-2148-14-5)
Supplement: Additional file 3: Table S3 — Summary of the partitions and models. The best-fit partitioning scheme and model according to PartitionFinder. [file 1471-2148-14-5-S3.pdf]

Table 3. **Summary of the partitions and models.** The best-fit partitioning scheme and model according to Partition Finder.

	Best model	Fragments in partition
1.	GTR+I+G	16S
2.	GTR+I+G	28S, CAD pos1, CAD pos2, COII pos1, COII pos2, COI 5´pos1, COI 5´pos2, COI 3´pos1, COI 3´pos2, H3 pos1, H3 pos2, Wnt pos1, Wnt pos2,
3.	HKY+G	COII po3 , COI 5´pos3, COI 3´pos3
4.	GTR+I+G	CAD pos3, H3 pos3, Wnt pos3
